# A new density-modification procedure extending the application of the recent |ρ|-based phasing algorithm to larger crystal structures

**DOI:** 10.1107/S2053273321004915

**Published:** 2021-06-21

**Authors:** Jordi Rius, Xavier Torrelles

**Affiliations:** a Institut de Ciència de Materials de Barcelona, CSIC, Campus de la UAB, Bellaterra, Catalonia 08193, Spain

**Keywords:** *S_M_* phasing algorithm, *ipp* procedure, |ρ|-based phasing residual, direct methods, origin-free modulus sum function, structure solution

## Abstract

The incorporation of the new peakness-enhancing fast Fourier transform compatible *ipp* procedure (*ipp* = inner-pixel preservation) into the recently published *S_M_* algorithm based on |ρ| [Rius (2020). *Acta Cryst* A**76**, 489–493] improves its phasing efficiency for larger crystal structures with atomic resolution data. Its effectiveness is clearly demonstrated via a collection of test crystal structures (taken from the Protein Data Bank) either starting from random phase values or by using the randomly shifted modulus function (a Patterson-type synthesis) as initial ρ estimate. It has been found that in the presence of medium scatterers (*e.g.* S or Cl atoms) crystal structures with 1500 × *c* atoms in the unit cell (*c* = number of centerings) can be routinely solved. In the presence of strong scatterers like Fe, Cu or Zn atoms this number increases to around 5000 × *c* atoms. The implementation of this strengthened *S_M_* algorithm is simple, since it only includes a few easy-to-adjust parameters.

## Abstract 2

The insertion of a peakness-enhancing fast Fourier transform compatible module in the novel *S_M_*,_|ρ|_ phasing algorithm improves its efficiency for larger crystal structures as shown with a collection of representative X-ray diffraction data sets taken from the Protein Data Bank.

## 1. Introduction   

The novel 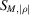 phasing function is rooted in the *Z_R_* origin-free modulus sum function, a nearly 30 years-old direct-methods phasing function (Rius, 1993 ▸). Both mainly differ in (i) the introduction of ‘Fourier transform’ calculations instead of the complex manipulation of ‘structure invariants’ (Rius *et al.*, 2007 ▸); (ii) the replacement of 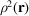 by 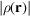 at each point **r** of the unit cell by using the property that 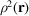 and 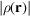 are positive-definite functions with similar shape (Rius, 2020 ▸). The resulting 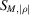 phase refinement function is defined by

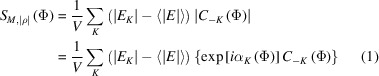

in which the *K* sum extends over all reflections (*i.e.* strong and weak ones), |*E_K_* | denotes the experimental structure-factor modulus with 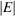 being their average value, *V* is the volume of the unit cell, and Φ denotes the collectivity of φ phases involved in the computation of ρ. The *C_K_* (Φ) = |*C_K_* (Φ)| exp[*i*α_*K*_(Φ)] complex quantity is the Fourier transform of the |ρ(Φ)| density function in terms of the 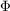 structure-factor phases to be refined. Their refinement is achieved by maximizing 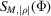 through the iterative 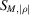 fast Fourier transform (FFT) algorithm. This algorithm has been developed in *P*1, since this symmetry is advantageous to *ab initio* phase refinements (Sheldrick & Gould, 1995 ▸). (Mathematically, however, nothing prevents its implementation as a full-symmetry algorithm.) As demonstrated by Rius (2020 ▸), maximizing *S_M_*_,|ρ|_ is equivalent to minimizing the phasing residual

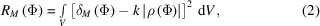

which measures the discrepancy between δ_*M*_(Φ) and |ρ(Φ)|. In integral (2), δ_*M*_(Φ) and *k* are, respectively, the inverse Fourier transform of (|*E_K_* | − 〈|*E*|〉) exp[*i*α_*K*_(Φ)] and a suitable scaling constant (Rius, 2012 ▸). Since integral (2) can be exactly worked out in terms of 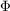 , its minimum value should correspond (for data reaching atomic resolution) to the true solution or an equivalent, to the maximum of the correlation coefficient

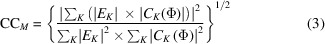

measuring the agreement between experimental and calculated modulus functions. CC_*M*_ rapidly increases at the beginning of the iterative 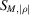 phase refinement, gradually stabilizes as it progresses and suddenly increases at the end (normally by 0.035–0.045 in just a few cycles) indicating that convergence has been attained.

One common feature of most iterative phase refinement algorithms working at atomic resolution and alternating between real- and reciprocal-space calculations is the density modification of the intermediate Fourier maps. Peak-picking is the simplest procedure which has been applied in the *Shake-and-Bake* approach (Weeks *et al.*, 1993 ▸; Miller *et al.*, 1993 ▸), *i.e.* once the centers and heights of the *N* highest peaks in the map have been determined (*N* is the expected number of non-H atoms in the unit cell) these are used to calculate the new structure-factor estimates. For large structures, however, application of the FFT algorithm (Cooley & Tukey, 1965 ▸) to the Fourier map is more efficient than direct calculation of the structure factors. In the literature other density-modification procedures can be found, *e.g.* in *SIR2000* the density fraction above a 2.0–2.5% threshold is kept in each map inversion, the rest set to zero [Burla *et al.* (2000 ▸) and Shiono & Woolfson (1992 ▸) for a related procedure]; Caliandro *et al.* (2008 ▸) have later shown the convenience of increasing this threshold when the resolution of the data is poorer than atomic. Also highly effective but more complicated is the density-modification scheme incorporated in *ACORN2* (Dodson & Woolfson, 2009 ▸). Alternatively, peakness in the electron-density function can be enhanced by multiplying it with a mask having unit Gaussians only at the previously determined peak positions (the rest being zero). This modification is part of Sheldrick’s intrinsic phasing procedure (Sheldrick, 2015 ▸) and allows the posterior application of the FFT algorithm. In the present work, the alternative peakness-enhancing *ipp* procedure (*ipp* = inner-pixel preservation) is described. It directly operates on the η = δ_*M*_ *m*_ρ_ product function of the *S_M_* algorithm wherein 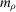 is the mask relating 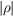 to 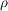 through the expression

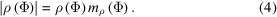

According to Rius (2020 ▸), the values of *m_ρ_* are 1 (for ρ > 0), 0 (for ρ between 0 and −*t*_ρ_σ_ρ_) and −1 (for ρ < −*t*_ρ_σ_ρ_) with 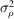 being the variance of ρ(Φ) and *t*_ρ_ ≃ 2.5. Hereafter 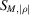 will be shortened to *S_M_* for simplicity.

## 2. The *S_M_* phasing algorithm with enhanced peakness: the *ipp* procedure   

**Figure 1:**
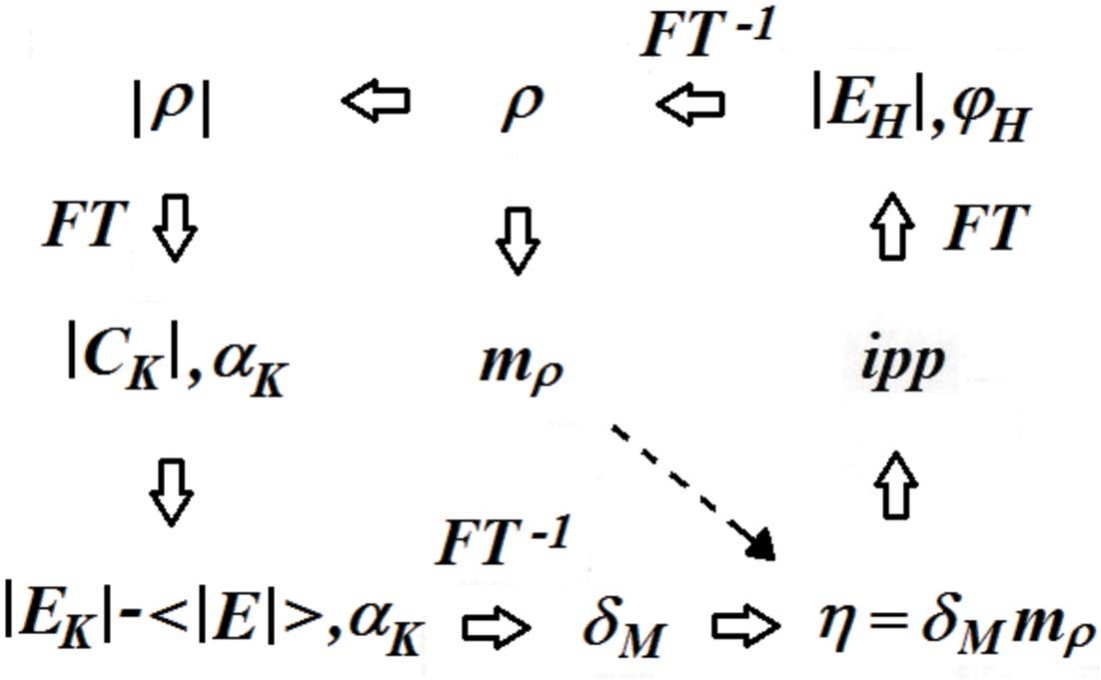
The recursive *S_M_*-*ipp* phase refinement algorithm with enhanced peakness: (upper right corner) φ phase estimates (either initial or updated values) are combined with experimental |*E*|’s to obtain ρ, |ρ| and *m*_ρ_ (the latter is stored). Next, the Fourier transform of |ρ| is calculated leading to new |*C*| and α values, and the former are used in the calculation of CC_*M*_. The new α values are combined with the experimental (|*E*| − 〈|*E*|〉) (lower left corner), and their inverse Fourier transform, δ_*M*_, is calculated. In the next step, function δ_*M*_ is multiplied with the stored *m*_ρ_ mask to give the η product function. Peakness in η is enhanced by applying the *ipp* density-modification procedure and, finally, the Fourier transform of the modified η supplies the updated φ phases. [Initial sets of φ estimates investigated in this article are either Φ_rnd_ (random phase values) or Φ_*M*′_ (phase values corresponding to the Fourier coefficients of *M*′, *i.e.* the randomly shifted modulus function).]

The phasing residual (2) can be minimized with the *S_M_* algorithm (Rius, 2020 ▸), *i.e.* by the iterative application of the modified tangent formula

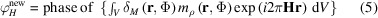

which corresponds to the angular part of the Fourier transform within brackets. One characteristic of the *S_M_* algorithm is the presence of the η = δ_*M*_ *m*_ρ_ product function. To enhance the peakness of η, the simple *ipp* procedure based on the preservation of the inner-peak pixels has been added to *S_M_*, giving rise to the *S_M_*-*ipp* algorithm (Fig. 1 ▸). This procedure consists of two well differentiated parts:

(i) Peak search in the η product function. The lowest value of η which is accepted as a peak is fixed by the *t*_η_ σ_η_ threshold ( 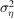 is the variance of η, and *t*_η_ a parameter allowing tuning of the threshold and normally ranging between 3.5 and 4.0). The η peaks are searched by looking for the density values of all 26 nearest grid points around a given central pixel (satisfying the above threshold criterion). This (*x*_o_, *y*_o_, *z*_o_) central pixel is considered a η peak if its density value is larger than the values of all its 26 nearest neighbor pixels, *i.e.* 8 (*x*_o_ ±1, *y*_o_ ±1, *z*_o_ ±1); 4 (*x*_o_, *y*_o_ ±1, *z*_o_ ±1); 4 (*x*_o_ ±1, *y*_o_, *z*_o_ ±1); 4 (*x*_o_ ±1, *y*_o_ ±1, *z*_o_); 2 (*x*_o_, *y*_o_, *z*_o_ ±1); 2 (*x*_o_, *y*_o_ ±1, *z*_o_); 2 (*x*_o_ ±1, *y*_o_, *z*_o_) (Rollet, 1965 ▸). If this is the case, the density value and the pixel coordinates of the central pixel are stored. At the end, the *N*_η_ stored peaks are ordered in decreasing strength. (Note, *t*_η_ and *N*_η_ are inversely related.)

(ii) Density modification of η. If *N*_η_ > *N*, then for each one of the *N* highest-ranked η peaks, the density values of the 26+1 inner-peak pixels are preserved. The density-modification procedure finishes by setting to zero all pixels of η not having preserved density values. For *N*_η_ ≤ *N*, the inner pixels of all *N*_η_ peaks will have preserved density values. The Fourier transform of the modified η yields the new φ estimates.

**Figure 2:**
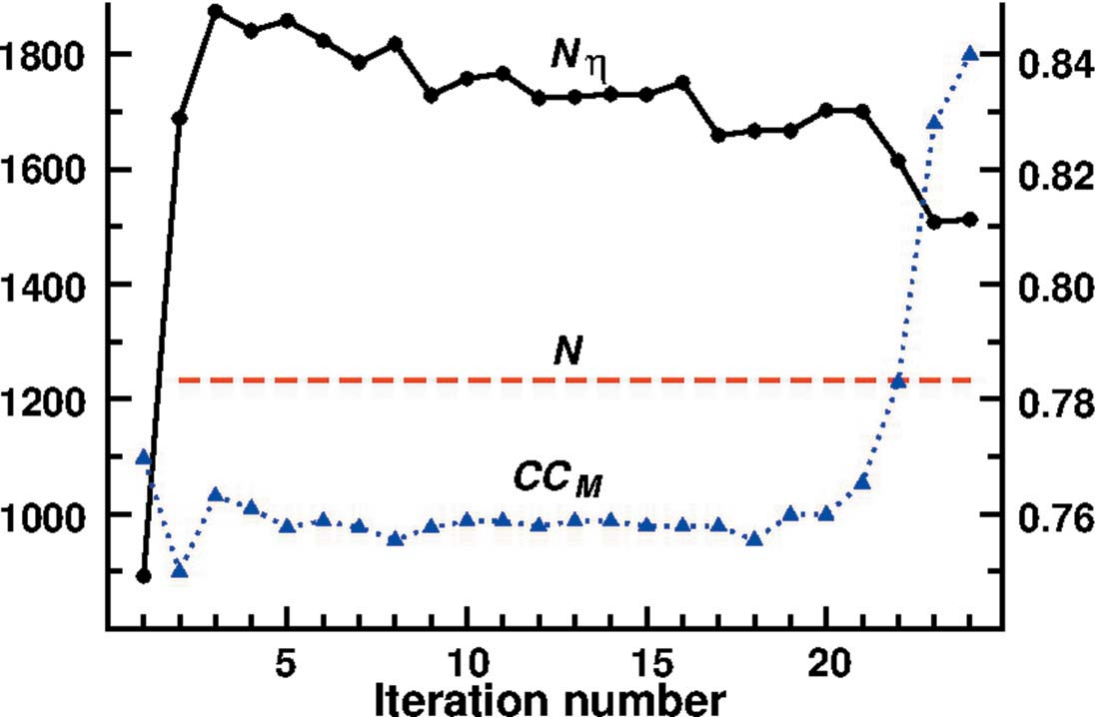
*S_M_*-*ipp* phasing with initial random phases (Φ_rnd_): variation of *N*_η_ and CC_*M*_ with the iteration number for data set 1a7z (*t*_η_ = 3.7). *N* is the number of non-H atoms in the unit cell*.*

Notice that accurate peak center positions are not necessary for the application of the *ipp* procedure; consequently, no peak interpolation is needed. Notice, also, that it is compatible with the ‘random omit maps’ strategy introduced in direct methods by Sheldrick (Usón & Sheldrick, 1999 ▸). For illustrative purposes, a successful *S*_*M*_-*ipp* phase refinement obtained with starting random (rnd) phases and with *t*_η_ = 3.7 is reproduced in Fig. 2 ▸. It is interesting to note that only *N*_η_(1) is smaller than *N* (the number 1 in parentheses indicates the iteration number).

Compared with the *S_M_* algorithm in Rius (2020 ▸) in which all reflections participate in the computation of the ρ synthesis, *S_M_*-*ipp* works better if ρ is calculated with only those *H* reflections for which |*E*| ≥ |*E*|_min_ with |*E*|_min_ ≃ 1.0, *i.e.* Φ only includes the large and moderate |*E*| values [however, the calculation of the δ synthesis remains unchanged, *i.e.* it extends to all *K* reflections (Fig. 1 ▸)]. Notice that the faster calculation of ρ in *S_M_*-*ipp* counteracts the extra computing time due to *ipp*. Concerning this point, a test performed on data set 1pwl showed that the duration of one iteration in *S_M_*-*ipp* and in *S_M_* is very similar. The *S*_*M*_-*ipp* algorithm has been programmed in a modified version of the *XLENS*_v1 code (Rius, 2011 ▸). In the test calculations, *N* always includes, besides the number of protein atoms, the number of solvent ones, *i.e.* water molecules.

## 3. The modulus function as initial estimate of ρ   

It is clear that the phasing process not only depends on the phasing algorithm but also on the starting phase values. In Rius (2020 ▸), the *S*_*M*_ algorithm was only tested by assigning random values to the initial phases, Φ_rnd_ = {φ_rnd_}. However, the ideal situation for any phasing algorithm is to start with phase values derived from initial ρ estimates (ρ_ini_) containing structural information. Since the *M* modulus synthesis is a Patterson-type synthesis (Ramachandran & Raman, 1959 ▸), it can be regarded as the sum of *N* weighted shifted images of the crystal structure (or its enantiomorph) (Wrinch, 1939 ▸; Buerger, 1950 ▸). Consequently, it contains valuable structural information and can be taken as ρ_ini_. The success of the phasing process will obviously depend on the capability of the phasing algorithm to develop one incomplete shifted image of the crystal structure while (gradually) suppressing the rest (working in *P*1 allows selection of one arbitrary image). The phasing process is greatly facilitated by the presence of a reduced number of strong scatterers in the unit cell with their corresponding images standing out from the rest [this justifies the separate treatment in the test calculations of compounds with weak, medium (atoms with *Z* < 19) and strong scatterers (*Z* ≥ 19)]. In multisolution phasing methods, each phase refinement trial requires a different ρ_ini_. This can be achieved by shifting the experimental *M* by a randomly generated **u** = **OO**′ vector to obtain the correspondingly shifted *M*′ function (*O* and *O*′ are the respective origins). The Fourier coefficients of *M*′ are 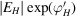 with 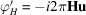 and Φ_*M*′_ = { 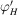 }. In this way each trial follows a different refinement path (in the test calculations, the sequence of **u** vectors is the same for all data sets). The number of selected phase refinement trials (*N*_trials_) is either 5, 25 or 50 depending on the success rate; the maximum number of allowed iterations per trial is always *N*_iter(max)_ = 1000 (excepting 3bcj with 200).

## 4. Comparison of the phasing efficiencies of the *S_M_-ipp* and *S_M_* algorithms   

The efficiencies of the *S_M_*-*ipp* and *S_M_* algorithms have been calculated for both Φ_rnd_ and Φ_*M*′_. For simplicity, the various phase refinement strategies are specified by A1, A2, B1, B2, *i.e.* A1: Φ_rnd_ with *S_M_*-*ipp*; A2: Φ_rnd_ with *S_M_*; B1: Φ_*M*′_ with* S_M_*-*ipp*; B2: Φ_*M*′_ with *S_M_*.

The compounds participating in the test calculations are listed in Tables 1 ▸ and 2 ▸. For those compounds in Table 1 ▸ only containing weak scatterers, the checked strategies are A1, A2 and B1 (Table 3 ▸). In the case of compounds with medium/strong scatterers (Tables 1 ▸ and 2 ▸), the investigated strategies are B1 and B2 (Tables 4 ▸, 5 ▸ and 6 ▸). To make comparisons between strategies stricter, corresponding refinement trials started with the same set of randomly generated phase values.

### 4.1. Compounds with only weak scatterers   

The data sets used in the tests of crystal structures with only weak scatterers are 1a7y, 3sbn, 1ob4, 1a7z and 1alz (Table 1 ▸). The first three data sets belong to small crystal structures and the last two to relatively large ones. Of these, 1a7z corresponds to a Cl-containing compound with 1228 atoms in the unit cell. In spite of the presence of Cl, it has been included in this section because the refinement protocol deposited in the Protein Data Bank (PDB) indicates that one Cl is partially occupied and the other has a rather large *B* value, so that their scattering powers are considerably reduced. The last data set (1alz) corresponds to the notoriously difficult crystal structure of gramicidin with 1348 C, N and O atoms in the unit cell and with nearly 25% of the atoms showing positional disorder.

**Figure 3:**
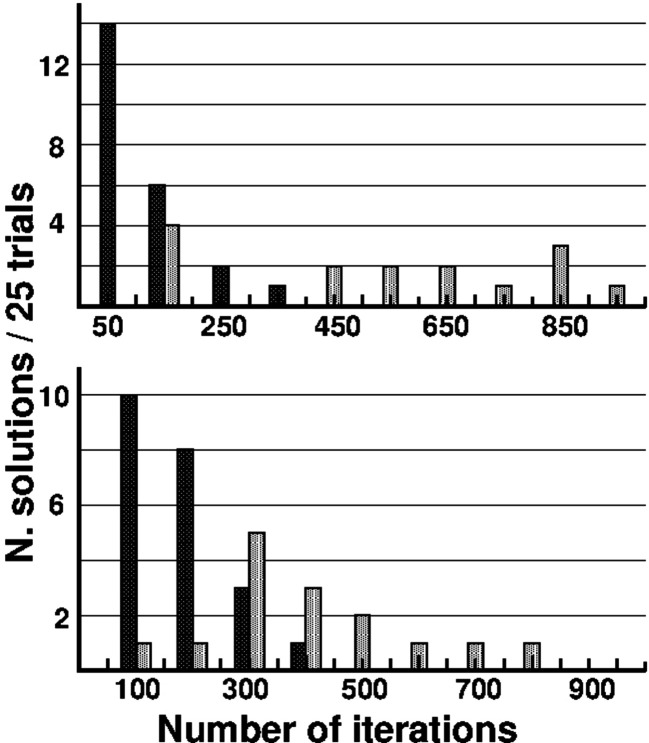
Effect of the *ipp* procedure on the phasing efficiency of the *S_M_* algorithm with Φ_rnd_. The two selected data sets belong to: (top) 3sbn (trichovirin) with 444 atoms in the unit cell; (bottom) 1a7z (Actino Z3) with 1228. True solutions obtained with/without the *ipp* procedure in black/gray (same starting random phase values for each pair of trials).

Of the two A1 and A2 phasing strategies, the best one is A1 (Table 3 ▸). Compared with A2, A1 yields the smallest 〈*N*_iter_〉 values and the largest number of successful trials for all five tested data sets, *i.e.* the correct solutions are found much faster when *ipp* is applied. The faster convergence of A1 is illustrated in Fig. 3 ▸ for data sets 3sbn and 1a7z. In the case of gramicidin, two correct solutions are obtained with A1 (trial 21 with *N*_iter_ = 136 and trial 45 with *N*_iter_ = 520) which represents one solution every 2.5 h using a desk computer (3.4 GHz); however, with A2 no correct solution was found. Regarding the A1 and B1 strategies, inspection of Table 3 ▸ indicates that A1 converges somewhat faster than B1 and is superior in the case of gramicidin (B1 gives no correct solutions).

### 4.2. Crystal structures with only medium scatterers   

The application of strategy B1 to ten compounds containing medium scatterers (1byz, 2erl, 1p9g, 3nir, 1a0m, 4lzt, lf94, 1hhu, 3odv and 3psm) is summarized in Table 4 ▸. In most cases (nine out of ten) phase refinements performed smoothly, *i.e.* all five trials converged. Of these nine cases, only 1a0m (conotoxin) required more iterations. The acquisition of the conotoxin data with a Cu rotating anode at room temperature (outermost shell is 1.10–1.14 Å) surely contributes to the different behavior of this data set. In contrast to the nine preceding cases, application of *S*_*M*_-*ipp* to 1f94 (bucandin) was less successful. Consequently, *N*_trials_ was increased to 25 to estimate more reliably the success percentage (32%). This structure has large atomic disorder (*B*_Wilson_ = 14.3 Å^2^) which is reflected in the large fraction of unobserved data in the 1.06–1.02 Å interval, *i.e.* 0.50 with *I* > 2σ(*I*). The influence of *ipp* on the phase refinement accuracy can be estimated with ΔCC_*M*_, *i.e.* the difference between CC_*M*_ values for *S_M_*-*ipp* and for *S_M_*. As can be clearly seen in Tables 3 ▸ and 4 ▸, ΔCC_*M*_ is only slightly negative, generally between −0.02 and −0.03, which suggests that truncation of the outer-peak regions during the application of the *ipp* procedure is not critical.

To estimate the influence of *ipp* on the convergence of the phase refinement, the same tests carried out with strategy B1 were repeated with B2 (Table 4 ▸). Comparison of both sets of *N*_iter_ values confirms the much faster convergence of B1.

### 4.3. Crystal structures with strong scatterers   

From Table 5 ▸ it follows that for compounds with heavy atoms of the first transition series, application of the B1 strategy allows the routine determination (in a reduced number of iterations) of crystal structures with *N* up to ≃5000 × *c* (*c* = number of centerings) provided that the data are of good quality and that at least the scattering power of one of the heaviest atoms is not weakened. The resulting 〈*N*_iter_〉 values go from 10 to 60 except for data sets 41au, 1pwl, 1heu and 1c7k for which it is larger. In the case of 41au the increase can be related to two of the three symmetry-independent seleno­methio­nine Se atoms showing partial occupancies, *i.e.* (0.52, 0.48) and (0.31 and 0.69) (Fanfrlik *et al.*, 2013 ▸). For 1pwl and 1heu, the larger 〈*N*_iter_〉 values could be ascribed to the larger *d*_min_ values (Table 2 ▸). For comparison purposes, the results obtained with strategies B1 and B2 are summarized in Table 6 ▸. Its inspection confirms the clear superiority of B1 over B2, especially for the larger test crystal structures.

## 5. Discussion   

One characteristic of the *S_M_* algorithm is its mathematical simplicity, a consequence of the straightforward implementation of the modified tangent formula (5). One relevant parameter of *S_M_* is *t*_ρ_ which modifies the threshold value in the calculation of |ρ| through expression (4). The value of *t*_ρ_ mainly depends on the scattering power of the strongest scatterer present in the crystal structure. In Rius (2020 ▸), *t*_ρ_ was found to be close to 2.5. In the current work, the test examples extend to a larger variety of structures in which the strongest scatterer can be weak, medium or strong. Respective *t*_ρ_ values giving satisfactory results have been found to be ≃2.5, ≃2.6 and ≃2.8.

Regarding the *ipp* procedure, its application requires the approximate knowledge of *N* and the estimation of *t*_η_. The *N* value used in the test calculations is the sum of both protein and solvent atoms (taken from the PDB), *i.e.* *N*_Prot_ + *N*_H2O_. An idea of 〈*N*_H2O_〉 can be obtained by averaging (*N*_Prot_ + *N*_H2O_)/*N*_Prot_ over all structures with more than 700 atoms listed in Tables 1 ▸ and 2 ▸ which gives 1.22 (5), *i.e.* 〈*N*_H2O_〉 ≃ 0.22 × *N*_Prot_. The second parameter, *t*_η_, controls the number of η peaks above the *t*_η_ σ_η_ threshold. It can be estimated from *Q* = *N*_η_(2)/*N*. Suitable *t*_η_ values are those for which *Q* is close to 1 or not much smaller (the *ipp* procedure does not use *N*_η_ peaks exceeding *N*). According to Tables 3 ▸, 4  ▸ and 5 ▸, values of *t*_η_ from 3.5 to 4.0 give *Q* values ranging from 1.5 to 0.7. Whatever the initial phase values may be, a successful refinement ends with a sudden increase of CC_*M*_ concomitant with a marked *N*_η_ decrease.

**Figure 4:**
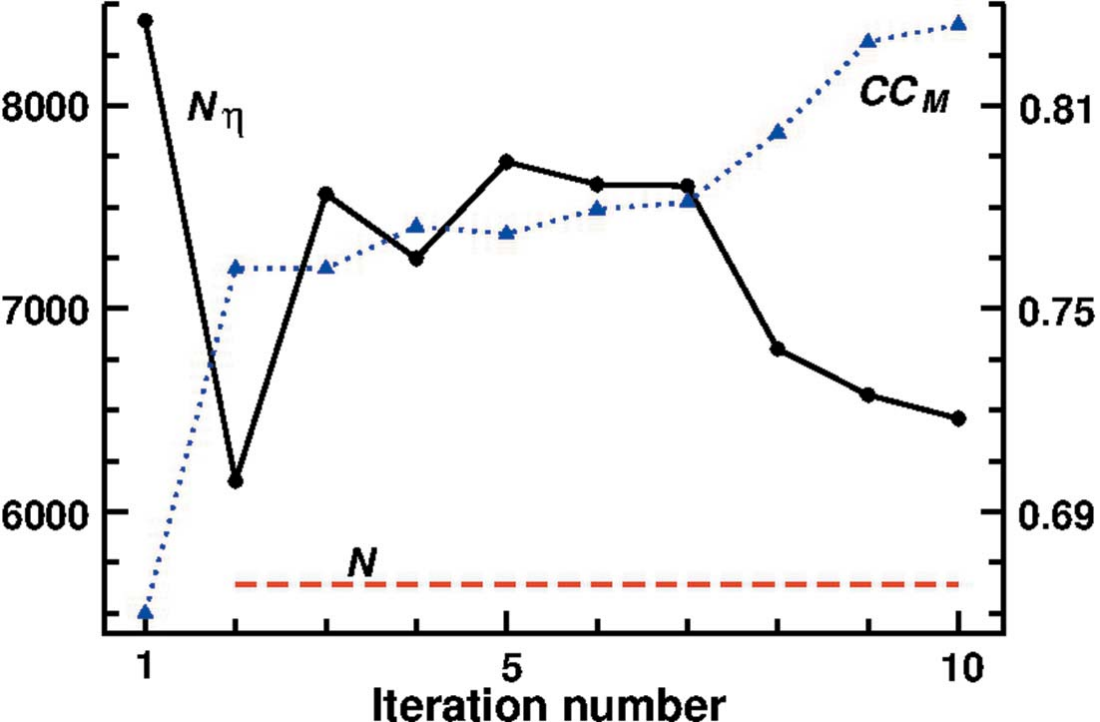
S*_M_*-*ipp* phasing with Φ_*M*′_: variation of *N*_η_ and CC_*M*_ with the iteration number for data set 3ks3 (*t*_η_ = 3.9). *N* = number of non-H atoms in the unit cell.

Of interest is the comparison of the *N*_η_(1) values obtained with strategies A1 (Φ_rnd_) and B1 (Φ_*M*′_) by using similar *t*_η_ values. As was already shown in Section 2, *N*_η_(1) is smaller than *N* for Φ_rnd_ (Fig. 2 ▸). However, for Φ_*M*′_ (Fig. 4 ▸), *N*_η_(1) is much larger than *N*, since here η essentially corresponds to the shifted modulus function with weakened origin peak. In the test calculations, the Φ_*M*′_ set at the end of the first iteration is always calculated with the *N* largest η peaks. The only exception is 1b0y. Since the unit cell of this compound contains four dominant scattering units (Fe_4_S_4_ clusters), only the 240 (= 16^2^ − 16) strongest η peaks (mostly corresponding to Fe–Fe interactions) were used.

**Figure 5:**
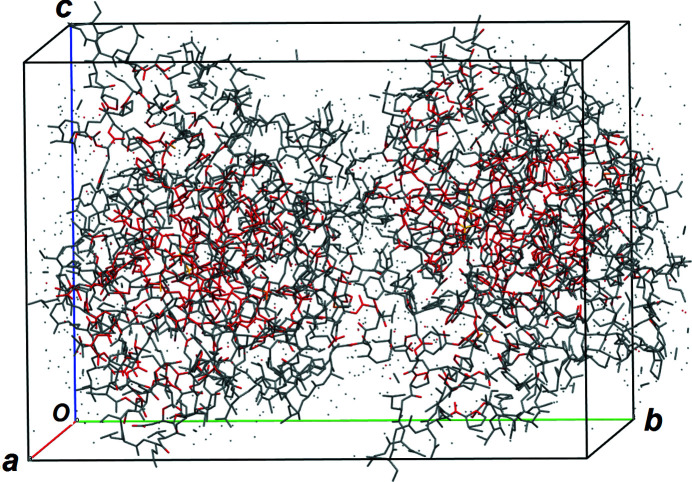
Unit-cell content of aldose reductase (Zhao *et al.*, 2008 ▸; data set 3bcj) showing the two unique protein chains related by the screw axis along *b* as obtained with the *S_M_*-*ipp* phasing algorithm directly from the experimental modulus synthesis (Φ_*M*′_) by assuming *P*1 symmetry (S and light atoms are found simultaneously). Atoms with higher refined peak strength are shown in red.

For the compounds in Table 1 ▸ (except for 3bcj), the average strength of the S/Cl peaks in the Fourier map is 30 (5) a.u. (a.u. = arbitrary units). For 3bcj, however, the strength increases to 59 a.u. The explanation for the much larger peak strength has to be sought in the ultra-high resolution of the experimental data favored by its lower measurement temperature (15 K compared with the usual 100 K). This test structure was selected to check the phasing capability of *S*_*M*_-*ipp* with ultra high resolution data. With 5934 atoms in the unit cell (solvent atoms excluded) this crystal structure is in the same order of magnitude as those listed in Table 2 ▸ containing strong scatterers. Application of *S*_*M*_-*ipp* with Φ_*M*′_ (strategy B1) yields success percentages of 80%, 36% and 0% for *d*_min_ = 0.78, 0.85 and 0.90 Å, respectively (Fig. 5 ▸ reproduces the *E* map of one arbitrary successful refinement). Notice that *S*_*M*_-*ipp* solves here the protein structure in one stage, *i.e.* it is not necessary to first locate single S atoms as, *e.g.*, done by McCoy *et al.* (2017 ▸).

A limitation of *S_M_*-*ipp* (when used as an *ab initio* phasing algorithm) arises for crystal structures belonging to high-symmetry point groups and having large asymmetric units, since then *N* becomes exceedingly large. Normally, the usual way to cope with such situations is to derive the initial Φ from a larger structure model by using, among others, molecular replacement or anomalous dispersion techniques. In such cases *S_M_*-*ipp* will become the phase refinement stage of a more general two-stage strategy.

## 6. Conclusions   

It has been shown that the introduction of the new peakness-enhancing *ipp* procedure in the *S_M_* phase refinement algorithm significantly improves the algorithm efficiency for diffraction data at atomic resolution and, consequently, has been incorporated as the default option. For *ab initio* structure determinations with *S_M_*-*ipp*, the proper choice of the type of starting phases is important. Regarding this point, the following rules could be established on the basis of the test calculations:

(*a*) For very small light-atom crystal structures either Φ_rnd_ or Φ_*M*′_ phases can be used (peak overlap in the modulus function can still be managed by *S_M_*-*ipp*).

(*b*) Starting with Φ_rnd_ is appropriate for crystal structures containing only weak scatterers (the largest *N* value tested is around 1500 atoms).

(*c*) Starting with Φ_*M*′_ is the best option for crystal structures with medium scatterers like S or Cl (largest *N* for routine determinations is 1500 × *c*). If no trial converges in *N*_iter(max)_ iterations, then phase refinement with Φ_rnd_ should be tried (with a larger *N*_iter(max)_); however, Φ_*M*′_ should always be the first choice.

(*d*) Use of Φ_*M*′_ is the best choice for crystal structures with strong scatterers. For metals belonging to the first transition series like Fe, Cu and Zn, the largest *N* value for routine determinations has been estimated to be about 5000 × *c* atoms (tests performed on data sets collected at ≃100 K). One characteristic of successful phase refinements starting with Φ_*M*′_ is their fast convergence. This allows one to reduce *N*_iter(max)_ and, consequently, increase the number of explored trials.

Finally, some words regarding data completeness are in order. As already mentioned in Section 1, the *S_M_* algorithm relies on the validity of the *R_M_* residual (2) which assumes that δ and ρ are proportional (which is satisfied for data sets reaching atomic resolution as is the case with the test calculations described in this work). If the intensities of the outer reflection shells are unobserved (a common situation for protein crystals), *R_M_* is no longer strictly fulfilled. Extrapolating the structure factors of unobserved reflections beyond the experimental resolution limit, *e.g.* by Fourier inversion of a suitably modified map, could be a solution for extending the applicability range of *R_M_* to moderate-resolution data sets. This ‘structure-factor extrapolation’ technique (Caliandro *et al.*, 2005*a* ▸,*b* ▸, 2007 ▸; see also Jia-xing *et al.*, 2005 ▸) is particularly effective for crystal structures containing heavy atoms (Caliandro *et al.*, 2008 ▸; Burla *et al.*, 2012 ▸). The combination of *S_M_* with the extrapolation technique could represent a further source of progress.

## Supplementary Material

The output of the test calculations A1_A2_B1_weak, B1_medium, B2_medium, B1_strong and B2_strong. DOI: 10.1107/S2053273321004915/ik5001sup1.zip

## Figures and Tables

**Table 1:** Data sets from the Protein Data Bank (PDB) used to compare the *S*_*M*_-*ipp* and *S*_*M*_ phasing algorithms corresponding to compounds with only weak scatterers (top five) or with weak and medium scatterers (remaining) Residues = number of residues; *c* = number of centerings; *N* = number of non-H atoms in the unit cell (PDB); *M* and H_2_O = number of medium scatterers and refined water molecules; %Sol = solvent volume percentage; *d*_min_ = minimum *d* spacing in Å of used reflection data; *T* = data collection temperature in K. (1a7y, 1ob4, 1a7z, 1alz, 2erl, 1a0m are rotating-anode or sealed-tube data sets; otherwise synchrotron data.)

PDB code	Compound	Residues	Space group	*N*/*c*	*M*/*c*	H_2_O/*c*	%Sol	*d*_min_	*T*
1a7y	Actino D^(1)^	33	*P*1	314	–	44	18	0.94	133
3sbn	Trichovirin^(2)^	30	*P*2_1_	444	–	32	24	0.95	100
1ob4	Cephaibol A^(3)^	17	*P*2_1_ *2*_1_ *2*	548	–	60	22	1.00	100
1a7z	Actino Z3^(1)^	22	*P*2_1_2_1_2_1_	1228	8Cl	4	49	0.95	173
1alz	Gramicidin A^(4)^	34	*P*2_1_2_1_2_1_	1348	–	4	30	1.00	120
1byz	Alpha1-peptide^(5)^	52	*P*1	479	1Cl	30	27	0.91	100
2erl	Er-1 pheromone^(6)^	40	*C*2	656	14S	44	20	1.00	273
1p9g	Antifungal^(7)^	41	*P*2_1_	702	20S	122	23	1.00	283
3nir	Crambin^(8)^	48	*P*2_1_	902	12S	196	31	1.00	100
1a0m	Conotoxin^(9)^	34	*I*4	1144	40S	168	24	1.10	286
4lzt	Lysozime^(10)^	129	*P*1	1183	10S	139	32	1.00	295
1f94	Bucandin^(11)^	63	*C*2	1232	20S	236	35	1.02	100
1hhu	Balhimycin^(12)^	28	*P*2_1_	1310	16Cl	250	22	0.89	100
3odv	Kaliotoxin^(13)^	76	P\bar 1	1392	32S	180	20	1.00	100
3psm	Plant defensin^(14)^	94	*P*2_1_	1882	16S	366	45	0.98	100
3bcj	Aldose reductase^(15)^	316	*P*2_1_	7308	26S	1374	43	0.78	15
					+ 3P			0.85	

(1) Schäfer *et al.* (1998 ▸); (2) Gessmann *et al.* (2012 ▸); (3) Bunkóczi *et al.* (2003 ▸); (4) Burkhart *et al.* (1998 ▸); (5) Privé *et al.* (1999 ▸); (6) Anderson *et al.* (1996 ▸); (7) Xiang *et al.* (2004 ▸); (8) Schmidt *et al.* (2011 ▸); (9) Hu *et al.* (1998 ▸); (10) Walsh *et al.* (1998 ▸); (11) Kuhn *et al.* (2000 ▸); (12) Lehmann *et al.* (2002 ▸); (13) Pentelute *et al.* (2010 ▸); (14) Song *et al.* (2011 ▸); (15) Zhao *et al.* (2008 ▸).

**Table 2:** PDB data sets used to test the *S_M_*-*ipp* and *S_M_* phasing algorithms corresponding to compounds with strong scatterers Residues, space group, *c*, %Sol and *d*_min_ as in Table 1 ▸. *N* = number of non-H atoms in the unit cell (PDB); *M* and *S* = number of medium and strong scatterers; H_2_O = number of refined water molecules. Data sets 2bf9, 8rxn and 1c7k measured at room temperature; otherwise at 100 K.

PDB code	Compound	Residues	Space group	*N*/*c*	(*M*+*S*)/*c*	H_2_O/*c*	%Sol	*d*_min_
2bf9	aPP^(1)^	36	*C*2	768	2Zn	164	31	1.00
8rxn	Rubredoxin^(2)^	52	*P*2_1_	1010	12S+2Fe	204	35	1.00
1w3m	Tsushimycin^(3)^	132	*P*1	1276	10Cl+24Ca	191	35	1.00
2ov0	Amicyanin^(4)^	105	*P*2_1_	2060	6S+2P+2Cu	432	34	0.95
1c75	Cythochrome 553^(5)^	71	*P*2_1_2_1_2_1_	2660	12S+4Fe	500	38	0.97
3d1p	Transferase^(6)^	120	*C*2	2702	2S+2Cl+4Se	498	29	0.95
1pwl	Aldose reductase Br^(7)^	316	*P*1	3030	14S+3P+1Br	429	25	1.10
1a6m	Myoglobin^(8)^	151	*P*2_1_	3154	8S+2Fe	372	36	1.00
41au	Geodin^(9)^	161	*P*2_1_	3278	2Ca+ 6Se†	740	40	0.99
1eb6	Deuterolysin^(10)^	177	*P*2_1_	3300	12S+2Zn	518	39	1.00
1b0y	H42Q^(11)^	85	*P*2_1_2_1_2_1_	3348	36S+16Fe	824	30	0.90
1x8q	Nitro­phorin 4C^(12)^	184	*C*2	3662	10S+2Fe	720	24	0.90
2fdn	Ferredoxin^(13)^	55	*P*4_3_22	3964	128S+64Fe	768	35	1.00
3fsa	Azurin^(14)^	125	*P*2_1_2_1_2_1_	4488	36S+4Cu	856	38	1.00
1c7k	Endoprotease Zn^(15)^	132	*P*2_1_2_1_2_1_	4532	12S+4Ca+4Zn	464	37	1.00
3ks3	H. C. anhydrase II^(16)^	260	*P*2_1_	5626	6S+2Zn	962	41	0.95
1heu	L. A. de­hydrogenase^(17)^	748	*P*1	7618	58S+4Cd	1297	50	1.15

(1) Glover *et al.* (1983 ▸); (2) Dauter *et al.* (1992 ▸); (3) Bunkóczi *et al.* (2005 ▸); (4) Carrell *et al.* (unpublished); (5) Benini *et al.* (2000 ▸); (6) Nocek *et al.* (unpublished); (7) El-Kabbani *et al.* (2004 ▸); (8) Vojtěchovský *et al.* (1999 ▸); (9) Fanfrlik *et al.* (2013 ▸); (10) McAuley *et al.* (2001 ▸); (11) Parisini *et al.* (1999 ▸); (12) Kondrashov *et al.* (2004 ▸); (13) Dauter *et al.* (1997 ▸); (14) Sato *et al.* (2009 ▸); (15) Kurisu *et al.* (2000 ▸); (16) Avvaru *et al.* (2010 ▸); (17) Meijers *et al.* (2001 ▸).

† Four disordered Se positions.

**Table 3:** Application of the *S_M_-ipp* and *S_M_* algorithms to crystal structures only containing weak scatterers (A1, A2 and B1 phasing strategies) The *t*_ρ_ parameter controlling the threshold of the *m*_ρ_ mask is always 2.50. *N*/*c* as in Table 1 ▸; *N_p_* = number of peaks showing up in the final *E* map above the *n* σ_ρ_ threshold; CC_*M*_ = correlation coefficient between experimental and calculated modulus function; *N*_iter_ = number of iterations to achieve convergence (n.c. = no convergence in 1000 iterations); *t*_η_ is the parameter controlling the number *N*_η_ of strongest η peaks; *Q* = *N*_η_(2)/*N*.

PDB code	*N*/*c*	Phasing strategy	*N*_*p*_/*c* (*n*)	CC_*M*_	〈*N*_iter_〉 for 5, 25 or 50 trials	*t*_η_	*Q*
1a7y	314	A1	376 (1.1)	0.86	〈*N*_iter_〉 = 37 (25×)	4.0	1.0
		A2	363 (1.1)	0.86	〈*N*_iter_〉 = 105 (25×)	–	–
		B1	370 (1.1)	0.85	〈*N*_iter_〉 = 100 (25×)	4.0	0.9
3sbn	444	A1	449 (1.1)	0.82	〈*N*_iter_〉 = 112 (23×); n.c. (2×)	3.7	1.4
		A2	456 (1.1)	0.85	〈*N*_iter_〉 = 558 (13×); n.c. (12×)	–	–
		B1	456 (1.1)	0.82	〈*N*_iter_〉 = 139 (21×); n.c. (4×)	3.7	1.2
1ob4	548	A1	564 (1.1)	0.86	〈*N*_iter_〉 = 308 (10×); n.c. (15×)	3.7	1.1
		A2	573 (1.1)	0.87	〈*N*_iter_〉 = 392 (3×); n.c. (22×)	–	–
		B1	569 (1.1)	0.86	〈*N*_iter_〉 = 446 (8×); n.c. (17×)	3.7	1.0
1a7z	1228	A1	1279 (1.1)	0.83	〈*N*_iter_〉 = 133 (22×); n.c. (3×)	3.7	1.4
		A2	1372 (1.1)	0.85	〈*N*_iter_〉 = 334 (16×); n.c. (9×)	–	–
		B1	1281 (1.1)	0.83	〈*N*_iter_〉 = 338 (23×); n.c. (2×)	3.7	1.0
1alz	1348	A1	1308 (1.5)	0.84	136, 520; n.c. (48×)	3.8	1.2
		A2	–	–	n.c. (50×)	–	–
		B1	–	–	n.c. (50×)	3.8	0.9

**Table 4:** Application of the *S_M_-ipp* and *S_M_* algorithms to crystal structures with medium scatterers Upper and lower lines refer to phasing strategies B1 and B2, respectively (except for 3bcj). *N*, *M*, *c* as in Table 1 ▸; *N_p_* = number of peaks showing up in the final *E* map above the *n* σ_ρ_ threshold; CC_*M*_ = correlation coefficient between experimental and calculated modulus function; *N*_iter_ = number of iterations to achieve convergence (n.c. = no convergence in 1000 iterations); *t*_ρ_, *t*_η_ = parameters controlling, respectively, the threshold of the *m*_ρ_ mask and the number *N*_η_ of strongest η peaks; *Q* = *N*_η_(2)/*N*.

PDB code	*N*/*c* (*M*/*c*)	*N*_*p*_/*c* (*n*)	CC_*M*_	*N*_iter_ for 5 or 25 trials	*t*_ρ_	*t*_η_	*Q*
1byz	479 (1Cl)	520 (1.1)	0.86	46, 56, 102, 134, 146	2.65	4.0	0.9
		529 (1.1)	0.86	234, 356, 535; n.c. (2×)		–	–
2erl	656 (14S)	610 (1.1)	0.78	32, 77, 112, 251, 270	2.50	4.0	0.8
		703 (1.1)	0.83	330, 475, 731; n.c. (2×)		–	–
1p9g	702 (20S)	695 (1.4)	0.85	24, 29, 30, 31, 43	2.60	3.8	1.1
		769 (1.4)	0.86	156, 174, 279, 296, 409		–	–
3nir	902 (12S)	934 (1.1)	0.86	24, 38, 46, 98, 127	2.70	4.0	0.7
		921 (1.1)	0.88	211, 333, 666, 690; n.c. (1×)		–	–
1a0m	1144 (40S)	1124 (1.3)	0.83	93, 97, 110, 158,186	2.60	3.7	1.0
		1342 (1.3)	0.86	217, 344, 366, 510, 844		–	–
4lzt	1183 (10S)	1134 (1.5)	0.83	43, 47, 48, 49, 51	2.65	4.0	1.0
		–	–	n.c. (5×)		–	–
1f94	1232 (20S)	1160 (1.1)	0.81	108, 110, 111, 171, 189, 353, 834, 897; n.c. (17×)	2.50	3.8	1.1
		1230 (1.1)	0.83	342, 681; n.c. (23×)		–	–
1hhu	1310 (16Cl)	1360 (1.4)	0.82	〈*N*_iter_〉 = 117 (17×); n.c. (8×)	2.60	3.9	1.1
		1378 (1.4)	0.85	〈*N*_iter_〉 = 426 (12×); n.c. (13×)		–	–
3odv	1392 (32S)	1480 (1.0)	0.72	18, 23, 23, 27, 36	2.50	3.5	1.2
		1499 (1.0)	0.77	176, 249, 256, 290, 583		–	–
3psm	1882 (16S)	1854 (1.4)	0.78	23, 24, 27, 33, 45	2.60	4.0	0.9
		2132 (1.4)	0.82	309; n.c. (4×)		–	–
3bcj†	7308 (26S)	7222 (1.3)	0.81	〈*N*_iter_〉 = 73 (20×); n.c. (5×)	2.65	3.8	1.5
		7086 (1.3)	0.82	〈*N*_iter_〉 = 114 (9×); n.c. (16×)		3.8	1.4

† Upper and lower lines correspond to B1 at *d*_min_ = 0.78 and 0.85 Å, respectively. *N*_iter(max)_ = 200.

**Table 5:** Application of *S_M_-ipp* to crystal structures containing strong scatterers (S) (strategy B1) *N* = number of non-H atoms in the unit cell (PDB); *c* = number of centerings; *N*_*p*_, CC_*M*_, *N*_iter_, n.c., *t*_ρ_, *t*_η_ and *Q* as in Table 3 ▸. [*t*_ρ_ = 2.80 except for 1w3m (2.60), 3d1p (2.70), 1a6m (2.75), 41au (2.70) and 3fsa (2.70); 〈*B*_Wilson_〉 is 6.8 (1.1) Å^2^ with the extrema being 5.3 for 2fdn and 9.1 for 1eb6.]

PDB code	*N*/*c* (*S*/*c*)	*N*_*p*_/*c* (*n*)	CC_*M*_	*N*_iter_ for 5 trials	*t*_η_	*Q*
2bf9	768 (2Zn)	709 (1.1)	0.81	10, 11, 12, 13, 15	3.5	2.2
8rxn	1010 (2Fe)	905 (1.1)	0.83	14, 15, 17, 18, 22	3.5	1.4
1w3m	1276 (24Ca)	1275 (1.4)	0.81	30, 33, 37, 42, 80	4.0	1.1
2ov0	2060 (2Cu)	1990 (1.5)	0.84	14, 15, 16, 16, 29	4.0	0.9
1c75	2660 (4Fe)	2541 (1.4)	0.82	12, 13, 14, 16,16	4.0	1.1
3d1p	2702 (4Se)	2642 (1.1)	0.83	12, 12, 14, 15, 16	3.8	1.1
1pwl	3030 (1Br)	3123 (1.1)	0.83	32, 54, 57, 62, 149	3.8	1.2
1a6m	3154 (2Fe)	3203 (1.1)	0.83	28, 30, 31, 37, 48	4.0	0.8
41au	3278 (6Se†)	3440 (1.1)	0.85	41, 69, 79; n.c. (2×)	3.8	1.0
1eb6	3300 (2Zn)	3406 (1.1)	0.82	16, 18, 23, 24, 40	4.0	0.9
1b0y	3348 (16Fe)	3360 (1.3)	0.76	38, 39, 41, 53, 79	3.5	1.5
1x8q	3662 (2Fe)	3510 (1.5)	0.83	34, 36, 58, 64, 92	4.0	1.0
2fdn	3944 (64Fe)	3832 (1.3)	0.81	21, 21, 22, 23, 26	3.8	1.0
3fsa	4488 (4Cu)	4580 (1.5)	0.83	31, 39, 40, 44, 56	4.0	1.0
1c7k	4532 (4Zn)	4548 (1.3)	0.84	80, 96, 128, 202, 399	4.0	0.9
3ks3	5626 (2Zn)	5588 (1.2)	0.83	9, 10, 10, 10, 10	3.9	1.1
1heu	7618 (4Cd)	7603 (1.1)	0.82	35, 40, 42, 45, 176	3.9	1.1

† Four Se atoms are partially disordered.

**Table 6:** Comparison of strategies B1 and B2 when applied to crystal structures with strong scatterers (S) For B2, the individual *N*_iter_ values are given; for B1, 〈*N*_iter_〉 corresponds to *N*_iter_ values in Table 5 ▸. It is evident that B1 (using *ipp*) performs better than B2 in all cases. *N* = number of non-H atoms in the unit cell (PDB); *c* = number of centerings; 〈*N*_iter_〉 = average number of iterations to achieve convergence (n.c. = no convergence in 1000 iterations).

PDB code	*N*/*c* (*S*/*c*)	B1 strategy 〈*N*_iter_〉	B2 strategy *N*_iter_ for 5 trials
2bf9	768 (2Zn)	12.2 (5×)	29, 29, 36, 39, 55
8rxn	1010 (2Fe)	17.2 (5×)	44, 53, 54, 56, 61
1w3m	1276 (24Ca)	44.4 (5×)	97, 109, 118, 124, 134
2ov0	2060 (2Cu)	18.0 (5×)	52, 54, 61, 62, 86
1c75	2660 (4Fe)	14.2 (5×)	41, 51, 52, 57, 73
3d1p	2702 (4Se)	13.8 (5×)	35, 38, 44, 45, 47
1pwl	3030 (1Br)	70.8 (5×)	n.c. (5×)
1a6m	3154 (2Fe)	34.8 (5×)	n.c. (5×)
41au	3278 (6Se†)	63.0 (3×)	400, n.c. (4×)
1eb6	3300 (2Zn)	24.2 (5×)	69, 86, 109, 156, 289
1b0y	3348 (16Fe)	50.0 (5×)	210, 225, 243, 254,259
1x8q	3662 (2Fe)	56.8 (5×)	261, 317, 404, 961, n.c.
2fdn	3944 (64Fe)	22.6 (5×)	62, 77, 82, 93, 97
3fsa	4488 (4Cu)	42.0 (5×)	163, 288, 324, 413, n.c.
1c7k	4532 (4Zn)	181.0 (5×)	n.c. (5×)
3ks3	5626 (2Zn)	9.8 (5×)	36, 42, 45, 46, 48
1heu	7618 (4Cd)	67.6 (5×)	226, 259, 273, 534, n.c.

† Four Se atoms are partially disordered.
