# Supplementary figures and images for: A new density-modification procedure extending the application of the recent |ρ|-based phasing algorithm to larger crystal structures
Source: Acta Crystallogr A Found Adv. 2021 Jun 21;77(Pt 4):339–47. doi: 10.1107/S2053273321004915 (PMC8248888; doi:10.1107/S2053273321004915)

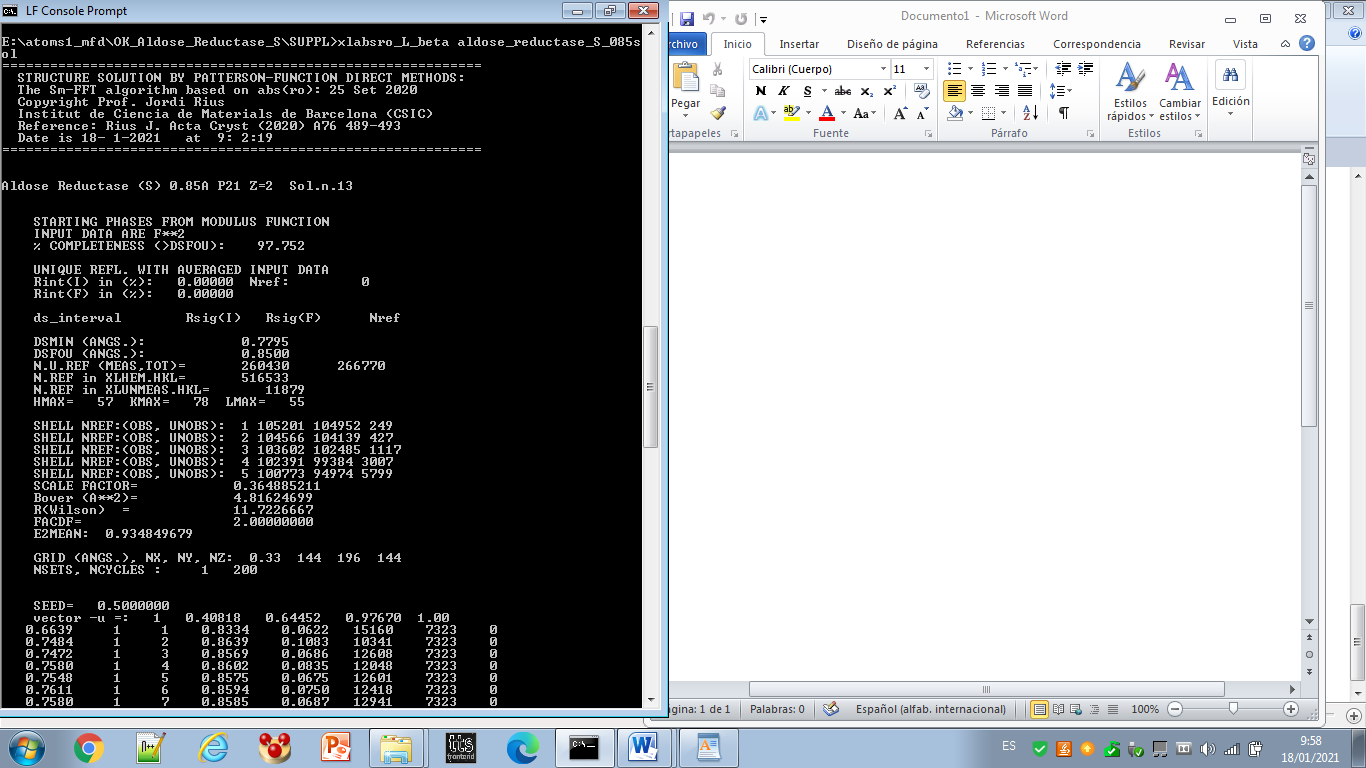


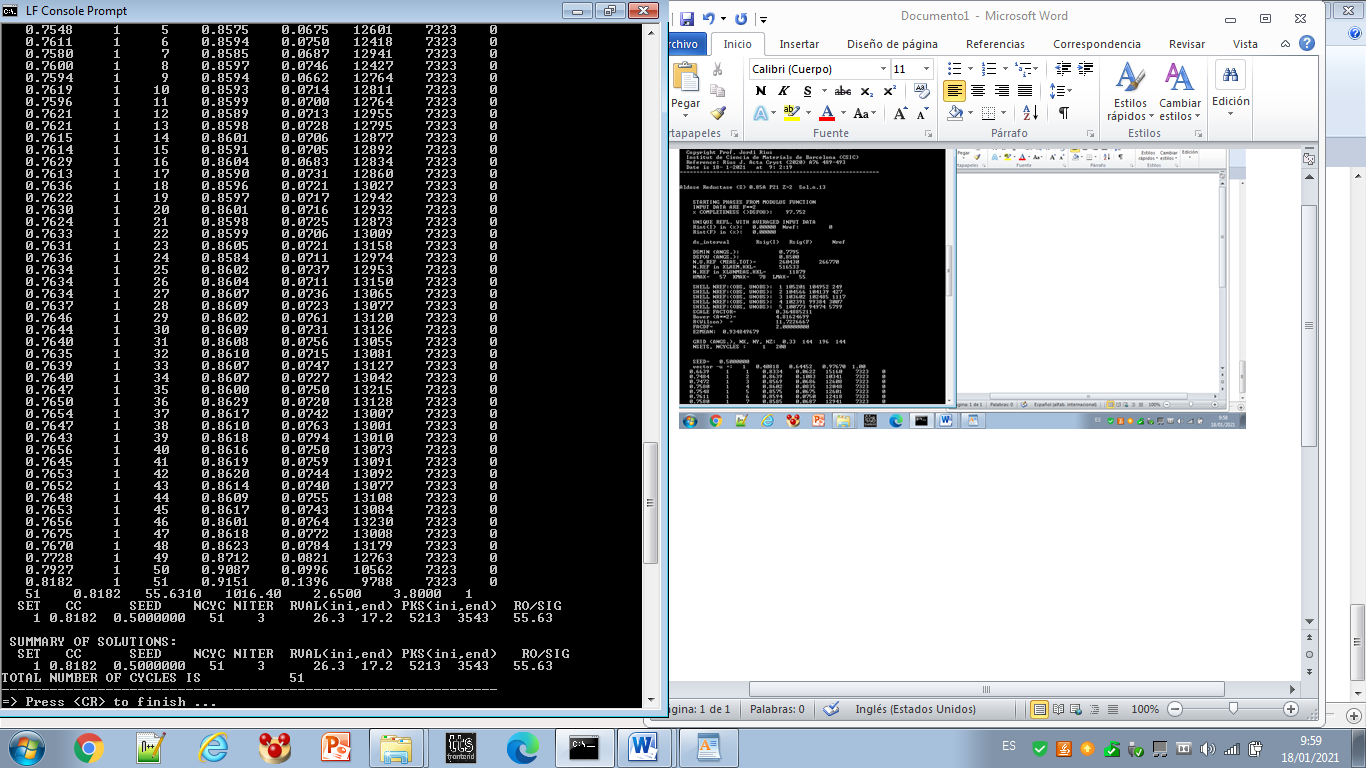

Supplement: Supplementary file 1 [file a-77-00339-sup1.zip › Suppl_material/B1_medium/sol_aldose_reductase_S_screen_085.docx]

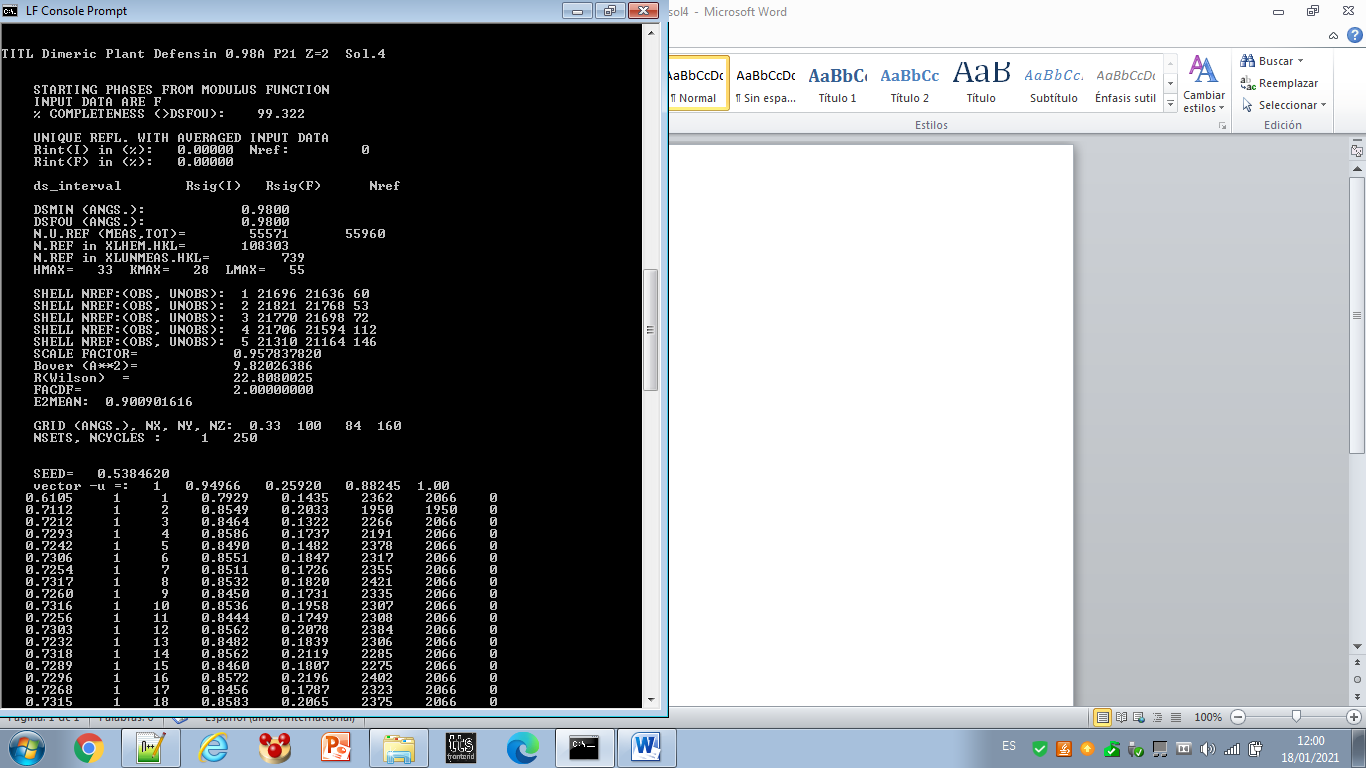


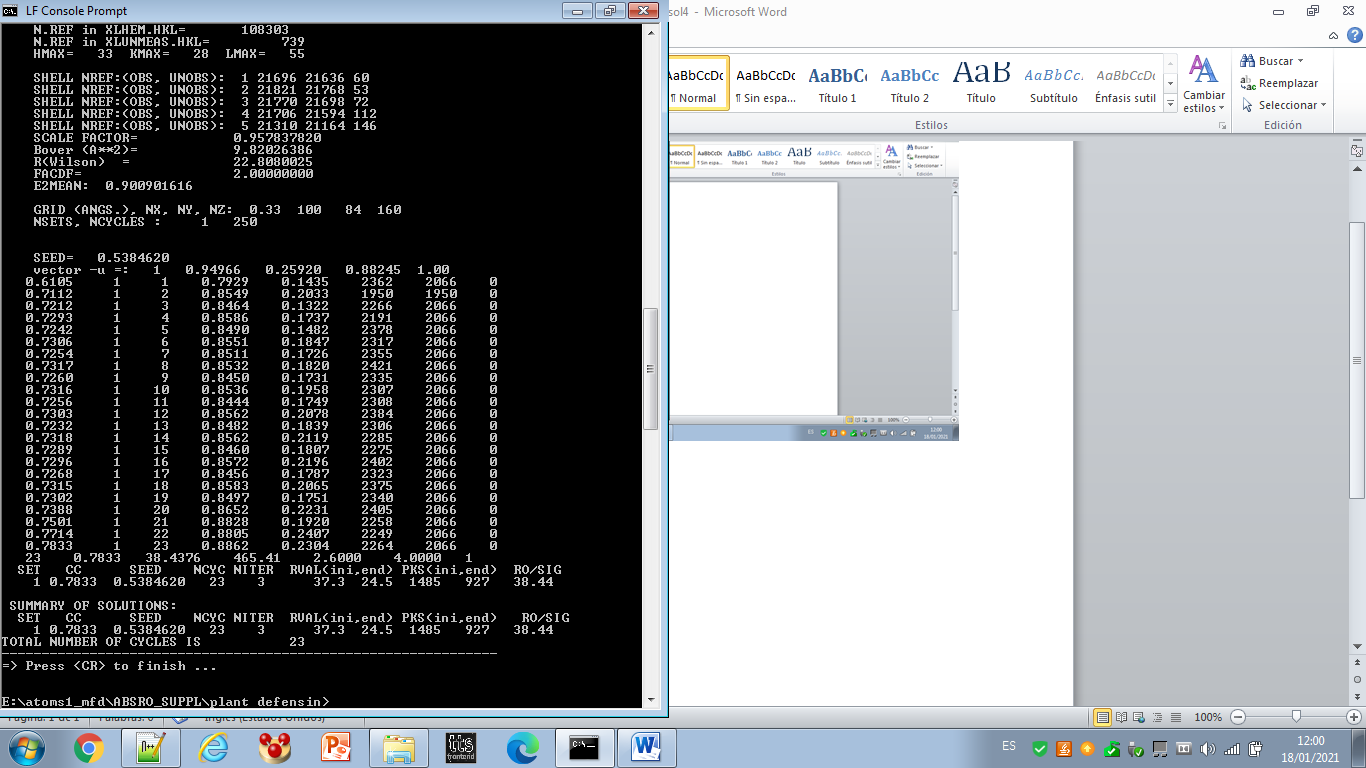

Supplement: Supplementary file 1 [file a-77-00339-sup1.zip › Suppl_material/B1_medium/sol4_defensin_screen.docx]

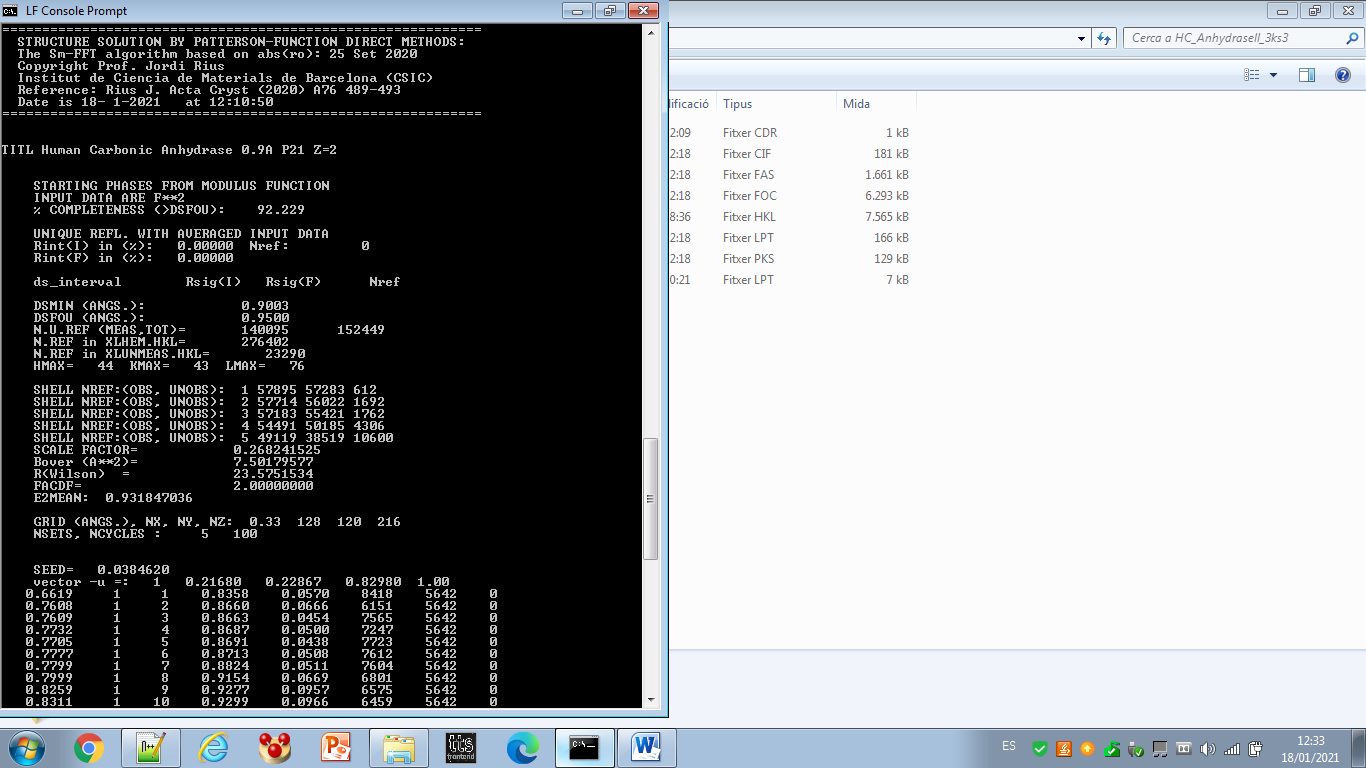


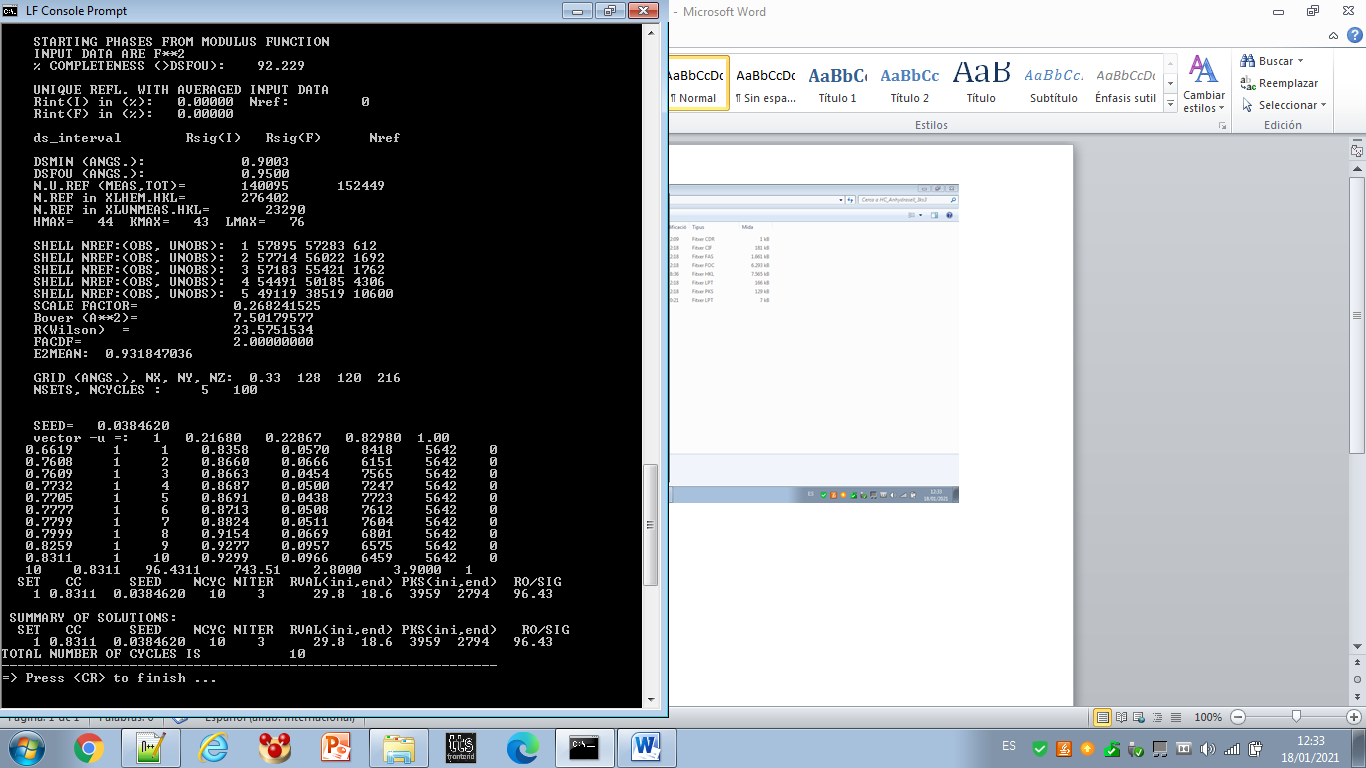

Supplement: Supplementary file 1 [file a-77-00339-sup1.zip › Suppl_material/B1_strong/sol1_anhydraseII_screen.docx]

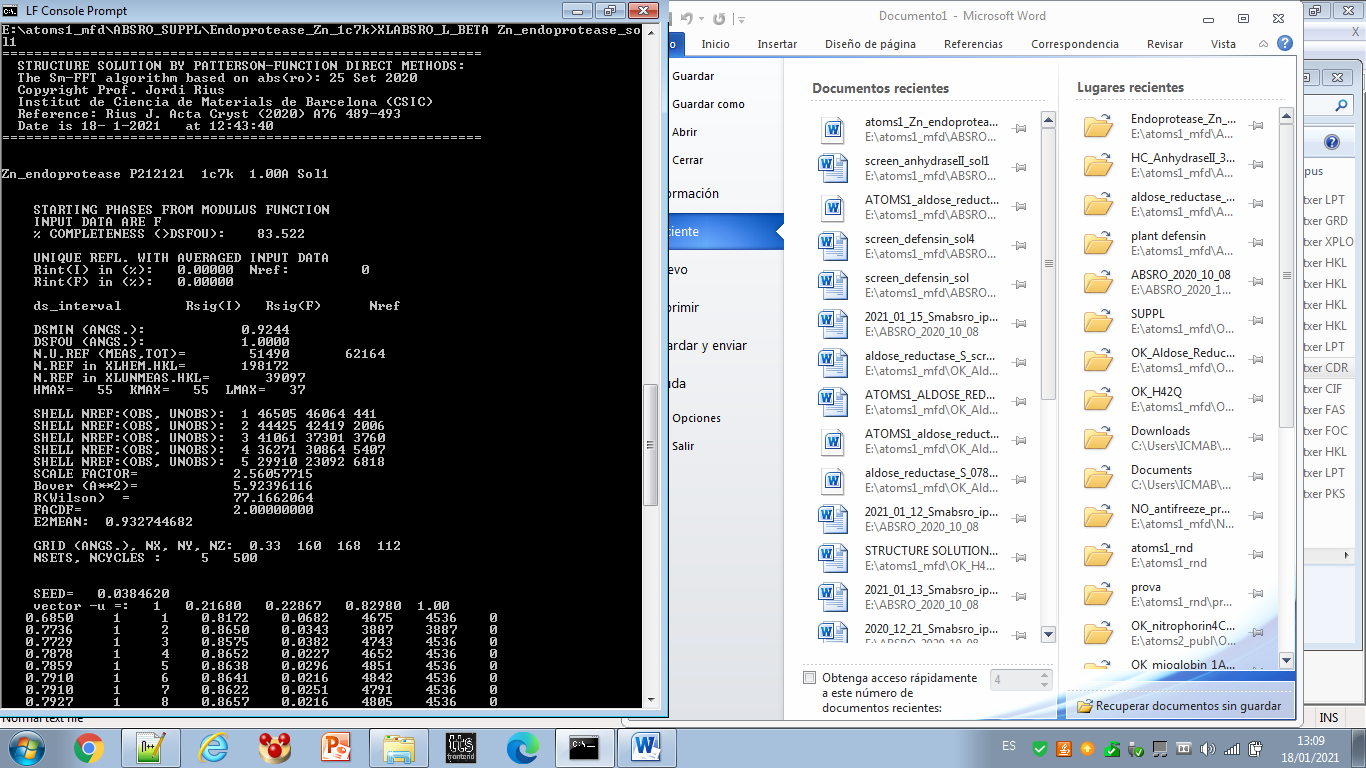


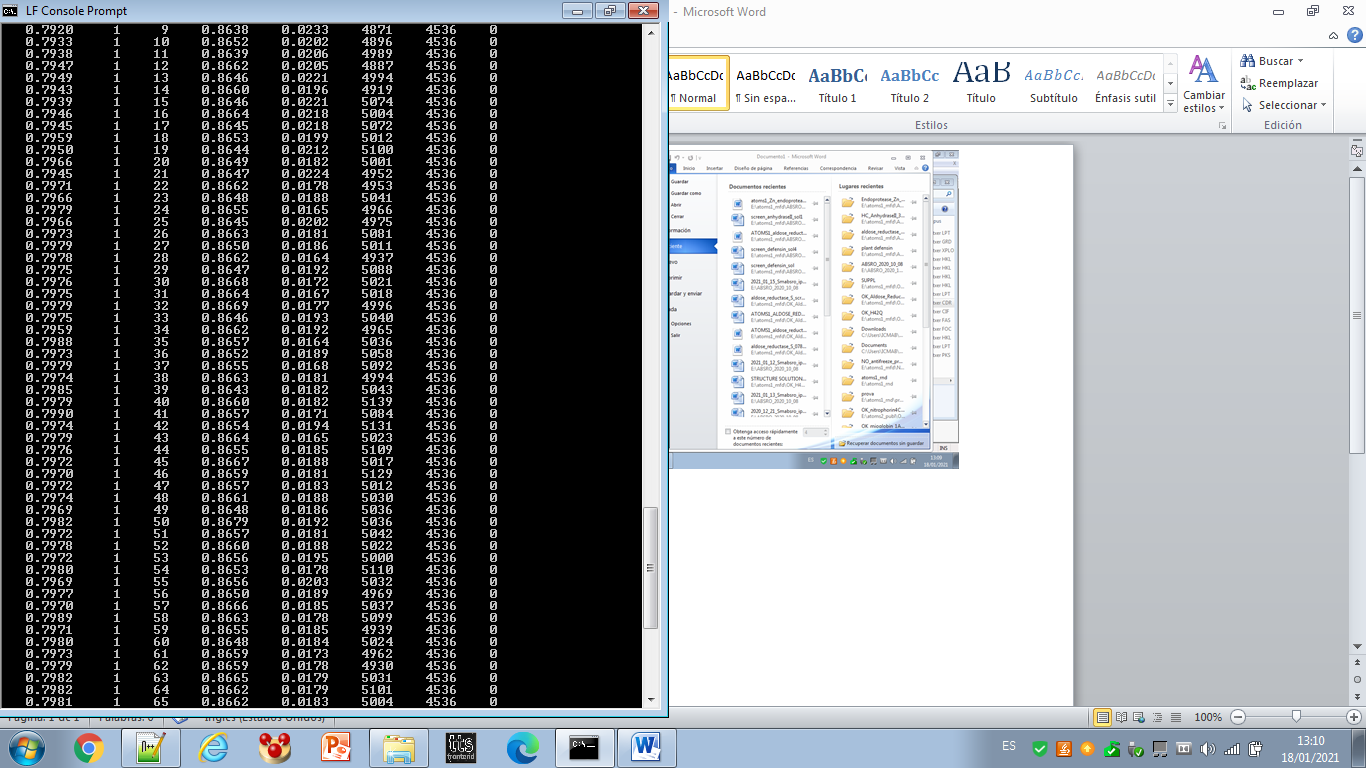

Supplement: Supplementary file 1 [file a-77-00339-sup1.zip › Suppl_material/B1_strong/sol1_Zn_endoprotease_sreen.docx]
